# Motivations for Alcohol Use among Men Aged 16–30 Years in Sri Lanka

**DOI:** 10.3390/ijerph6092408

**Published:** 2009-09-08

**Authors:** Bilesha Perera, Mohammad Torabi

**Affiliations:** 1 Department of Community Medicine, Faculty of Medicine, University of Ruhuna, Galle, Sri Lanka; E-Mail:pperera@indiana.edu; 2 Department of Applied Health Sciences, Indiana University, Bloomington, IN 47405, USA

**Keywords:** alcohol, motives, young males, Sri Lanka, stress

## Abstract

Psychometric properties of a new scale that measures motivations towards alcohol use were examined using a sample of 412 male alcohol users in Sri Lanka aged 16–30 years. In addition, associations between drinking motives and drinking frequency were explored. Confirmatory factor analysis showed that a 3-factor model consisting of the factors personal enjoyment, tension reduction, and social pressure fit the data well. Overall, tension-reduction motivation was found to be prominent in the context of young males’ drinking behavior in Sri Lanka. Associations between stress and alcohol use among young males warrant further investigations.

## 1. Introduction

In Sri Lanka, alcohol use is a growing public health issue [1–3], and, because it is a predominantly male habit there, alcohol use disproportionately harms Sri Lankan males. Adolescents and young adults in the country are highly vulnerable to the onset and continuation of the habit [4]. Beer is probably the most popular alcoholic beverage among Sri Lankan youth, and a significant surge in beer sales has been observed over the last few years [5–7]. In 2003, 19.3 million liters of beer were produced in the country and the corresponding figure for the year 2007 was 29.5 million liters, a 53 percent increase. Socio-cultural factors, such as urbanization and westernization and environmental factors, such as availability and affordability, may have contributed to this upward trend in the sale of alcohol.

The consumption patterns of alcohol by different age groups and the social-cognitive determinants of alcohol use, such as drinking motives, have not been clearly understood in the context of Sri Lanka. This is partly due to the widespread production of illegal liquor in a country where no records of the quantity and sale of illegal liquor are kept [8]. Another partial reason is the shortage of epidemiological and psycho-social data pertaining to the problem. Preventive programs targeted at young people should be based on a scientific knowledge of the distribution and determinants of alcohol use if they are to be effective in curbing the problem. Thus, there is a significant need to understand how and why young people in Sri Lanka use alcohol.

The reasons and motives for drinking alcohol are closely associated to the drinking patterns and consequences of alcohol use [9,10]. Different age groups may develop different motivations towards alcohol use that are shaped by many factors, including culture and environment [11,12]. Thus, motivation is considered a key concept in behavioral and psycho-social models of alcohol use. Since different drinking motives are associated with different types of drinking behavior [9,10] and culture plays a significant role in motivating or de-motivating people toward various behaviors, proper understanding of motives that direct young people to drink would help public health and education authorities formulate effective public health policies and develop cost-effective measures to curb the alcohol problem.

Researchers have identified a number of domains of drinking motives [9,10], and among them, personal enjoyment, social pressure, and tension reduction have been identified as prominent. Further, each domain is shown to be related to different aspects of alcohol use. For example, the personal enjoyment (enhancement) motive was found to be associated with heavy drinking, whereas social motives were identified to be associated with lighter drinking patterns and were more prevalent among young alcohol users [13,14]. Tension-reduction (anxiety reduction) motives were found to be related to solitary drinking and to problem drinking [13,15]. However, most of these studies have been conducted in the West, limiting the understanding of how these relationships work in non-western cultures. In the Sri Lankan context, there are certain culture-specific motivational factors of drinking behavior. Young males in Sri Lanka drink to become more prominent among peers and to attract females in social gatherings. Since the prevalence of alcohol use in the country is very low among women compared to men (5% verses 53%), alcohol use often symbolizes manhood, and thus, drinking behaviors are occasionally used by males to dominate family members and neighbors. A new scale was developed based on the existing drinking motives questionnaires [9,10,13] that incorporates some of these culture-specific motivational factors.

To date and to our knowledge, no studies have been conducted on the motivation toward alcohol use among people in Sri Lanka. This study’s purpose was to test the 3-factor structure of a scale developed to measure drinking motives and to examine the relationships between drinking motives and drinking patterns in a sample of males aged 16–30 years in Sri Lanka. It was hypothesized that the drinking motives scale consist of three factors: personal enjoyment, social pressure, and tension reduction. It was also hypothesized that drinking motives were related to drinking frequency.

## 2. Methods

A cross-sectional survey design was used. An anonymous, self-administered questionnaire was employed to collect data. Boards for the protection of human subjects from universities in both the United States and Sri Lanka approved the study protocol.

### 2.1. Sampling

Purposive sampling method was used to select sample subjects. Efforts were made to obtain an equal number of adolescents (16–19 years of age) and young adults (20–30 years of age) for the sample, so that the sample would represent the young males in the study population. Men aged 16–30 years, who were in the streets, universities and other educational institutes, in work places, or at homes during the survey period and who identified themselves as either occasional or regular drinkers, were invited to participate in the survey after explaining to them briefly the purpose of the survey and the anonymous nature of the data collection method. Those who agreed to participate in the study were surveyed using the questionnaire.

The survey was conducted in two settings: urban and semi-urban. Three medical undergraduate students were recruited and trained as data collectors. The people in the area respect and have a high confidence in those working or studying in the medical field, thus recruitment of medical students as data collectors increased the response rate. Only about 5% of the subjects who were asked to participate refused to do so. Data were collected for a period of two weeks in January 2007 and a total of 448 subjects participated in the study.

### 2.2. The Instrument

The survey instrument consisted of several variables: age, drinking frequency, and 20 questions on drinking motives. Drinking frequency of the participants was obtained using the item *I use alcohol* followed by four response categories: *daily*, *2–3 times a week*, *2–3 times a month*, and *2–3 times a year.* A literature survey was done and expert opinions were sought to identify motives towards alcohol use in young people in Sri Lanka [10,16–19]. The 3-dimensional drinking motives scale developed by Cooper, Russell, Skinner, and Windle (1992) was used as the base in the development of our new scale. In addition, ideas and opinions expressed on drinking motives by more than 100 young males, by whom the first author came into contact during his health education and prevention activities in the community, were also considered when selecting culturally-appropriate items for the scale. The scale developed by Cooper and colleagues (1992) has a total of 15 items and the new scale has 20 items. Some items from the scale developed by Cooper and colleagues, such as *I use alcohol because it helps me to forget my worries*, were included in the new scale. New culturally-appropriate items, such as *I drink alcohol because it will enhance my creative ability* and *I drink alcohol because it helps me to control others*, were added to the new scale. The new scale varies from the contemporary drinking motive scales that have been developed and used in the West.

A jury of experts consisting of a psychiatrist, two social scientists, and a psychologist examined the items for content validity. Based on their evaluation, the final scale of motive towards alcohol use was constructed using 20 rating items (see Table 1 for scale items). Four Personal Enjoyment (PE) items, 10 Tension-Reduction (TR) items, and six Social Pressure (SP) items were included in the scale. For example, *I drink alcohol because I like the taste* was a PE item, and *I drink alcohol because it is customary for men on special occasions* was a SP item. There were four response categories for each of these 20 items which were scored as follows: *to a greater extent* equals 3*; to some extent* equals 2; *to little extent* equals 1; and *not relevant* equals 0. The internal consistencies of the three factors using Cronbach’s alpha methods were as follows: alpha_PE_ = 0.48; alpha_TR_ = 0.74; and alpha_SP_ = 0.62. To assess the impact of each factor on drinking habits, each item in each of the three subscales were added up to obtain 3 subscale scores.

### 2.3. Data Analysis

The collected data were checked for consistency. Descriptive and bivariate analyses of the data set were done using SPSS 15.0 [20]. Confirmatory Factor Analysis (CFA) of the 20 item motives scale toward alcohol use was conducted using Lisrel 8.80 [21].

## 3. Results

After cleaning and consistency checking, analysis was done using 412 sample subjects. The age of the participants ranged from 16–30 years (*M = 20.27*, *SD = 3.63*). Forty-eight participants (11.7%) were married. Of the total, 102 (24.8%) had a job at the time of the survey and the others were either students or unemployed. Nine participants (2.2%) were daily users, 33 (8%) use alcohol 2–3 times a week, 76 (18.4%) 2–3 times a month and the remaining 294 (71.4%) 2–3 times a year. Means, standard deviations, and correlations among subscales and total scale are provided in Table 1.

Correlations among subscales and total scale ranged from 0.28 to 0.90 (*p < 0.01*). The strongest association was found between tension reduction and total score, and a relatively weaker association was found between personal enjoyment and social pressure.

### 3.1. Confirmatory Factor Analysis (CFA)

The 3-factor structure of the scale *motivations towards alcohol use* was tested using Lisrel 8.80. All indicators in the model had loadings above 0.40 and were significant at the *p < 0.01* level (Table 2). Loadings greater than 0.40 are generally considered as acceptable [22]. The overall fit of the model was acceptable: χ^2^ (167, *N* = 412) = 323.16; χ^2^/*df* ratio = 1.93:1; CFI = 0.98; and the RMSEA = 0.048 [23]. Thus, in this sample of alcohol users, motivations towards alcohol use can be divided into 3 factors: personal enjoyment motives, social pressure motives, and tension-reduction motives.

### 3.2. Relationships between Motivations and Drinking Habits

To test the second hypothesis, associations between the 3 motivational factors and drinking frequency were examined using multiple regression. This analytical method was chosen so that the effects of the drinking motive dimensions could be mutually adjusted. The results are presented in Table 3.

As can be seen from the individual beta weights, only tension reduction predicts drinking frequency. Thus, it is reasonable to infer that young males in Sri Lanka are less likely to be motivated to use alcohol because of its enhancement effect or because of the social pressure exerted by peer or social groups to use alcohol.

## 4. Discussion

The present study aimed to develop and test a 3-factor measurement of drinking motives. It also explored whether drinking motives were related to young males’ drinking frequency and, if so, which drinking motives were. As expected, according to CFA, the 3-factor model on drinking motives (i.e., based upon personal enjoyment, tension-reduction, and social pressure) complemented the data on this sample of male alcohol users aged 16–30 in southern Sri Lanka. Although similar instruments used in western countries [10,16,18,19] were employed in the development of the scale, the importance of the cultural embedding of drinking motives were also considered. Thus, it is safe to recommend that the scale can be used, with modifications if necessary, to examine drinking motives in other population groups across the country. However, future research should also consider use of drinking motives scales that are cross-culturally validated to compare results across countries. To our understanding, this study is the first to report on drinking motives of young males in Sri Lanka, a middle-income country in South Asia. Thus, undoubtedly, further research is needed to refine the scale and confirm the results.

Results of the study suggest that drinking to reduce tension seemed to be the most emphatic motive of alcohol use in this sample of young males. Stress, suicidal ideation, and suicide rates among young Sri Lankan people are ranked among the highest in the World [24,25], and the general belief is that alcohol helps to relieve distress. That climate may have motivated tension reduction via alcohol to become more prevalent in this young population. This warrants further research in the field.

In studies conducted in other countries, social motives were found to be associated with moderate alcohol use, and personal enjoyment (enhancement) motives with heavy drinking [15,16,26]. In this study, the quantity of alcohol used by the participants was not determined, so it was not possible to discriminate between moderate and heavy alcohol users in this sample. However, as has been demonstrated in other studies [3,27], most of the respondents in our sample should be either occasional or moderate drinkers. Heavy drinkers are generally found in the middle and older age groups in the context of people in Sri Lanka. Personal enjoyment and social pressure motives were found to be weaker motives of alcohol use compared to the tension-reduction motive in this study population. This may suggest that in countries like Sri Lanka, where the norm is abstinence from any type of alcoholic beverage, a relatively lower number of users consider alcohol as a pleasant drink. In fact, alcohol does not have a so called “rich, good taste,” and it is only a myth constructed and propagated by the alcohol industry [1]. Studies have shown that those who use alcohol to enhance their positive affect status often use alcohol alone and in settings such as bars [13,16]. Results of this study are consistent with this finding because drinking at bars or in college settings by young men is very rare behavior in Sri Lanka.

The cross-sectional design does not allow for any inference of a cause-effect relationship. Thus, relationships between motives and drinking patterns are better tested with longitudinal data. The use of purposive, non-probability sampling procedure may have limited the generalizability of the findings. However, it should be noted that in countries like Sri Lanka, where alcohol use is considered as “anti-social” behavior, especially by young people, refusing to participate and underreporting drinking behaviors would become major threats to selecting a representative sample if we were to select a random sample from households. Although self-report measures would raise questions of reliability and validity, the use of medical undergraduates in data collection and collecting data in non-threatening atmospheres, such as on the streets in most occasions, reduced this problem tremendously. It is also likely that some items in the scale may have related to more than one motivational factor, and items not included in this scale do have an impact on drinking motives. Further, alcohol habits should ideally be investigated using the type, quantity, and frequency of use. Future research needs to address these methodological and measurement issues.

## 5. Conclusions

The study advocates a 3-factor structure for a scale of motivations towards alcohol use, the scale developed and used for this study. However, the scale needs to be validated using a larger representative sample. Tension-reduction motivations seemed to be the most important social-cognitive factor in young Sri Lankan males’ drinking behavior. These findings have several implications for public health research and interventions. There is need for a continued focus on individual tension-reduction reasons for drinking in adolescents and young adults in substance use prevention programs. Alcohol motives are likely to have been shaped by other indirect and distal forces such as availability and the media. For example, television scenes that glamorize its use or incorporate strong symbolic meaning, such as rebellion against prejudice, while featuring alcohol may cause young people to initiate and continue drinking. Research that offers a better understanding of psycho-social and environmental factors associated with alcohol use behavior among the younger population in Sri Lanka is urgently needed.

## Figures and Tables

**Table 1.** Subscales and total scale of motivations towards alcohol use: Correlations, means and standard deviations.

**Motives**	**1**	**2**	**3**	***M***	***SD***

**1. Personal Enjoyment**	–	–	–	2.10	1.99
**2. Tension Reduction**	0.43	–	–	4.26	4.59
**3. Social Pressure**	0.28	0.45	–	2.54	2.78

**Total Score**	0.64	0.90	0.72	8.91	7.43

**Table 2.** Scale items and factor loading of the 3-factor model of motivations towards drinking.

**Item**	Factor Loading*

I use alcohol because,	

*Social Pressure*	
my friends drink	0.61
it is difficult to refuse	0.46
other people are drinking	0.77
it will enhance my creative ability	0.51
it is customary for men on special occasions I	0.59
want to be prominent	0.67

*Personal Enjoyment*	
I like the taste	0.62
it makes me feel good	0.71
I get thirsty	0.66
it goes well with the meals	0.41

*Tension Reduction*	
it helps me to relax	0.58
it would ease me when I get blamed	0.60
it helps me to sleep	0.61
it helps me to forget my worries	0.70
it helps me to get rid of restlessness and tense	0.61
it helps me to cheer up when I get dull or boring	0.67
it gives me energy	0.65
it is a habit	0.45
it helps me to face difficulties with confidence	0.72
it helps me to control others	0.44

* All items were loaded significantly on their respective factors, *p < 0 .01.*

**Table 3.** Multiple regression analysis predicting drinking frequency from 3 motives towards alcohol use.

**Drinking Motives**	**B**	***SE*B**	**β**

**Constant**	1.12	0.06	–
**Personal Enjoyment**	0.04	0.02	0.097
**Tension-reduction**	0.05	0.01	0.311*
**Social Pressure**	0.01	0.01	0.001

R^2^ = 0.13 (*ps* < 0.001),

* *p* < 0.001.
